# Modality Switching in Landmark-Based Wayfinding

**DOI:** 10.3389/fpsyg.2022.888871

**Published:** 2022-06-10

**Authors:** Mira Schwarz, Kai Hamburger

**Affiliations:** Department of Experimental Psychology and Cognitive Science, Faculty of Psychology and Sport Science, Justus Lieblig University, Gießen, Germany

**Keywords:** modality switch, switching costs, landmarks, olfactory, visual, wayfinding

## Abstract

This study investigates switching costs in landmark-based wayfinding using olfactory and visual landmark information. It has already been demonstrated that there seem to be no switching costs, in terms of correct route decisions, when switching between acoustically and visually presented landmarks. Olfaction, on the other hand, is not extensively focused on in landmark-based wayfinding thus far, especially with respect to modality switching. The goal of this work is to empirically test and compare visual and olfactory landmark information with regard to their suitability for wayfinding including a modality switch. To investigate this, an experiment within a virtual environment was conducted in which participants were walked along a virtual route of 12 intersections. At each intersection, landmark information together with directional information was presented, which was to be memorized and recalled in the following phase, either in the same or in the other modality (i.e., visual or olfactory). The results of the study show that, in contrast to the no-switching costs between auditory and visual landmarks in previous studies, switching costs occur when switching modality from visual to olfactory and vice versa. This is indicated by both longer decision times and fewer correct decisions. This means that a modality switch involving olfactory landmark information is possible but could lead to poorer performance. Therefore, olfaction may still be valuable for landmark-based-wayfinding. We argue that the poorer performance in the switching-condition is possibly due to higher cognitive load and the separate initial processing of odors and images in different cognitive systems.

## Introduction

Every day, people are challenged to get from their current location to a destination, whether it is finding their way home from a train station or just locating the nearest supermarket. Navigating through our environment thus represents an everyday task in human as well as animal life. Here, Montello (2005) makes a distinction between two components of navigation, which were also taken up by Montello and Sas (2006): Wayfinding and locomotion. Wayfinding is described as “the efficient goal-directed and planning part of navigation” (Montello and Sas, 2006, p. 2) and is therefore directly associated with problem solving. In addition, locomotion is the “real-time part of navigation” (Montello and Sas, 2006, p. 2), in which we try to avoid obstacles and arrive at our destination without further complications. In conclusion, navigation is a combination of wayfinding, i.e., route planning, which is the cognitive component, and locomotion, i.e., the process of moving along the route.

As soon as we are planning a route, we orientate ourselves on the basis of streets, buildings, or other objects (e.g., street signs, statues and the like). However, it is not just visual landmarks which play an important role even though research in landmark-based wayfinding mainly focusses on the visual aspects in human navigation (e.g., Lynch, 1960; Presson and Montello, 1988; Sorrows and Hirtle, 1999). Holden and Newcombe (2013) introduce a model of combining a variety of sources based on evidence concerning their validity. In this model, the reliability of spatial estimation accuracy increases when different modalities (i.e., auditory and visual cues) are combined in a Bayesian framework (Holden and Newcombe, 2013). The impact of non-visual elements coupled with visual elements on human spatial cognition has hardly been investigated. However, the explanatory approach of Holden and Newcombe (2013) was recently taken up by Siepmann et al. (2020) in a study indicating effects of sound positions in maps as cues for spatial memory performance.

Orientation by smell is mainly associated with species other than humans. In the animal kingdom, the ability to orientate by olfactory information has been demonstrated primarily in desert ants (e.g., Steck et al., 2009, 2011; Steck, 2012), rats (e.g., Rossier and Schenk, 2003) and dogs (Hepper and Wells, 2005; Reddy et al., 2022). Even untrained ring-tailed lemurs are able to track odor plumes, disproving the traditional belief that primates are unable to do so (Cunningham et al., 2021). Our own research has repeatedly addressed this bias towards vision in human spatial cognition research (e.g., Hamburger and Knauff, 2019) and demonstrated that humans are also able to orient themselves with auditory, visual verbal (i.e., words visually presented on screen) as well as olfactory cues (e.g., Röser et al., 2011; Hamburger and Röser, 2014; Karimpur and Hamburger, 2016; Hamburger and Knauff, 2019).

Apart from wayfinding research, several studies in other research fields are often concerned with switching costs. Switching costs, or more precisely within-task switching costs, are costs that arise when information from a certain task is presented to the user in a different sensory modality than expected (Kotowick and Shah, 2018). In addition to the within-task switching costs, there is also a cost for switching between tasks in which a different task has to be performed than the one that was initially learned (Arbuthnott and Woodward, 2002). This means that the modality remains the same, but the task changes. However, in wayfinding, information is not always available in the same modality in which we learned it (i.e., unimodal processing). So, what happens when the task stays the same (e.g., finding the correct path) but the modality switches (e.g., from visual to auditory information) within this task? What cognitive costs occur when we need to switch from one processing modality to another? In wayfinding research, it has been shown that there are no or hardly any switching costs in wayfinding performance (i.e., correct route decisions) when comparing visual and auditory landmark information within a wayfinding task (Hamburger and Röser, 2011). However, as mentioned above, olfactory information may also be of relevance and should not be underestimated (e.g., Hamburger and Karimpur, 2017). Are people able to alternate, i.e., switch modality, between vision and olfaction without additional cognitive costs, i.e., more time required or more errors? In the following, wayfinding with modality switches between visual and olfactory landmarks are compared to wayfinding without a modality switch. The results could be of interest especially in the field of interventions for elderly people and people with impaired vision, for whom it is necessary to deal with a specific modality, which is often required especially in unfamiliar environments (Hamburger, 2020).

People orientate themselves to their immediate environment in order to arrive at their destination. One core aspect in human orientation are orientation points, so-called landmarks (for review see Yesiltepe et al., 2021). A landmark is described by Lynch (1960) as any object that potentially serves as a reference point. Accordingly, a variety of different reference points can serve as landmarks, including trees, traffic lights, but also buildings or man-made objects (for an overview, see for example Lynch, 1960; Golledge, 1999).

The fact that landmarks can have a positive effect on wayfinding performance was shown by Sharma et al. (2017). In this study participants were given a wayfinding task that included a condition with and a condition without landmarks. The participants of the landmark condition made fewer mistakes and required less time on average compared to the participants of the condition without landmarks (Sharma et al., 2017).

The relevance of visual landmarks was demonstrated by, for instance, Denis et al. (2014) who compared routes with and without visual orientation points. Students learned either a route through an urban environment without visual references or a route in a neighborhood with many local stores and urban objects. Participants exposed to the landmark-rich environment with photographs of scenes along the route provided higher recognition scores and shorter decision times than participants who were not presented with visual references. In this case, visual landmarks had a positive impact on participants’ performance.

Human wayfinding with different sensory modalities than vision was tested by Hamburger and Röser (2014) who used different modalities to guide participants through a virtual maze. Their participants were divided into three experimental groups (visual, verbal or acoustic) and had to remember a route with the help of either visual, verbal or acoustic landmarks coupled with directional information. In the wayfinding phase, they had to indicate the correct direction at each intersection based on the landmark information given in the previous learning phase. Contrary to what might be expected, the participants showed a similar level of wayfinding performance for all three conditions. Visual, verbal, and acoustic information successfully constituted landmark information. Thus, human wayfinding can be supported not only through visual (e.g., Denis et al., 2014), but also non-visual landmark information (e.g., Hamburger and Röser, 2014).

This again supports the assumption that visual landmarks are not the only helpful means for finding one’s way. Therefore, other modalities should also be taken into account. Unfortunately, studies on human olfaction are rare in spatial cognition research. Nevertheless, to illustrate the current state of research on human wayfinding including olfactory landmarks we provide a few exceptions here. Dahmani et al. (2018) found an intrinsic relationship between olfaction and spatial memory which is probably rooted in the parallel evolution of the olfactory and hippocampal systems. Porter et al. (2007) found out that humans are able to follow a scent path just like rats and dogs do and are able to become better with practice. Furthermore, Jacobs et al. (2015) showed that humans are able to return to a previously learned location on a map with the help of olfactory cues only. This finding suggests that humans might use this odor-map as mechanism for navigation, too. An experiment by Hamburger and Knauff (2019) has shown that olfactory landmark information can be considered in the context of human wayfinding as well. They investigated this question in order to gain a more comprehensive understanding of the wayfinding ability of people with the help of olfactory information. In their study participants were walked through a virtual maze in which odors were presented as landmark information. At each intersection they had to memorize and later recall the olfactory information. It was demonstrated that participants were able to use the olfactory information to find their way (i.e., wayfinding performance was clearly above chance level). Further, olfactory landmarks have also been addressed in studies on how visually impaired people navigate in everyday life (Koutsoklenis and Papadopoulos, 2011).

Another relevant aspect regarding landmark-based wayfinding is modality switching and possibly associated switching costs. In the following, we refer to the within-task switching costs mentioned above (Kotowick and Shah, 2018) that arise when the task remains the same but the used modality changes (e.g., a picture of a clove of garlic is learned, but orientation must be based on the smell of garlic).

Hamburger and Röser (2011) dealt with the question of whether a modality switch between learning and recalling routes results in additional cognitive costs, i.e., more time required for the route decisions or more incorrect decisions. They contrasted different constellations of a modality switch of visual, acoustic and (visual) verbal landmarks. In the learning phase, either animal words, or sounds, or pictures had to be learned. In a subsequent wayfinding phase, introducing a modality switch or not (e.g., visual ➔ acoustic, visual ➔ visual), landmarks had to be recalled and with their help the way should be found. In none of the constellations additional switching costs occurred. Only the comparison of visual and (visual) verbal landmarks revealed differences in decision times.

Furthermore, Karimpur and Hamburger (2016) also investigated the wayfinding performance of participants using animal pictures and sounds. The difference to the previous study, however, was that they were not just concerned with unimodal but also multimodal processing. Similar wayfinding performances were found independent of whether participants were confronted with congruent stimuli (e.g., image of a dog paired with the barking of a dog) or incongruent stimuli (e.g., image of a dog paired with the chirping of a bird). Improved performance was demonstrated in the multimodal condition compared to the unimodal condition, which, according to Karimpur and Hamburger (2016), could be due to activation of both the visual and auditory sensory channels and therefore result in more elaborate representations or just better access to the stored information.

Kotowick and Shah (2018) also addressed the issue of modality switching. More specifically, they investigated the question of whether switching modality during navigation using navigation devices has certain advantages. They examined a system that switches between visual and haptic navigation guidance. Temporarily, performance deteriorated, but switching modalities seems to be beneficial for longer navigation tasks and to reduce both habituation effects and stimulus-specific adaptation.

In the following study, switching between visual and olfactory landmark information is contrasted with no-switch conditions in order to shed light on possible modality switching costs in landmark-based wayfinding.

Based on the theoretical and empirical background, it can be assumed that a modality switch is accompanied by none or marginal switching costs. However, it is important whether switching costs are defined as correct route decisions (i.e., correct turns) or as the time required for decision-making. The time required can be differentiated between the initial processing time and the time required to retrieve the correct route decision. Studies show that response times in the olfactory system range from 600 to 1,200 ms (Cain, 1976), which is significantly longer than the 200 ms interval observed for visual, auditory and tactile stimuli (Spence et al., 2000). People can respond to visual stimuli as early as 100 ms apart (Posner and Cohen, 1984), whereas the perception of odors is typically studied at 20–30 s intervals. The temporal resolution here is therefore 200 times greater for odor perception than for visual perception. The initial processing time is thus longer for olfactory than for visual inputs (Cain, 1976; Spence et al., 2000), whereas there should be little difference in the time required to retrieve the correct response, given the previous research in this area (e.g., Hamburger and Röser, 2011).

For this reason, it was hypothesized that a modality switch in the “switch” condition will result in (1) significantly higher decision times compared to the “no switch” conditions. Furthermore, based on the previous findings in other modalities [auditory, visual, and (visual) verbal] it was expected that the “switch” condition will result in (2) the same relative number of correct decisions compared to the “no switch” conditions. The experiment was based on a one factorial between-subjects design with four levels. The independent variable varied whether a modality switch occurred or not (“switch” vs. “no switch”). In the “no switch” condition, olfactory landmarks were presented to one group and visual landmarks to another in the learning and wayfinding phase. The “switch” condition was also divided into two groups that differed in the modality at learning and test (olfactory ➔ visual vs. visual ➔ olfactory). The dependent variables were the participants’ decision times on the one hand and the relative number of correct decisions on the other.

## Materials and Methods

### Participants

A total of 30 students (17 females and 13 males) of the Justus Liebig University were tested. The age range of the participants was 19–66 years (*M* = 24.80, SD = 8.53).

Exclusion criteria included any type of restriction in the ability to smell, such as respiratory problems or flu-like infections. Further exclusion criteria included epilepsy and non-corrected visual impairment. Participants were informed in advance to avoid spicy food and smoking on the day before the experiment, as this could have impaired the ability to smell. In addition, the participants were not supposed to use perfume before and during the experiment to ensure that no distraction due to additional odors occurred. Participation was voluntary and was compensated with course credits if required. All participants were naïve with respect to the research question and provided informed written consent prior to participation. The study was approved by the local ethics committee (Department of Psychology, JLU; 2014-0017).

### Material

The program OpenSesame 3.2.8 (Mathôt et al., 2019), was used to present routes with 12 orthogonal intersections and for data recording. In total, there were three different route sequences. Participants were pseudo-randomly assigned to the different experimental conditions.

**Figure 1:**
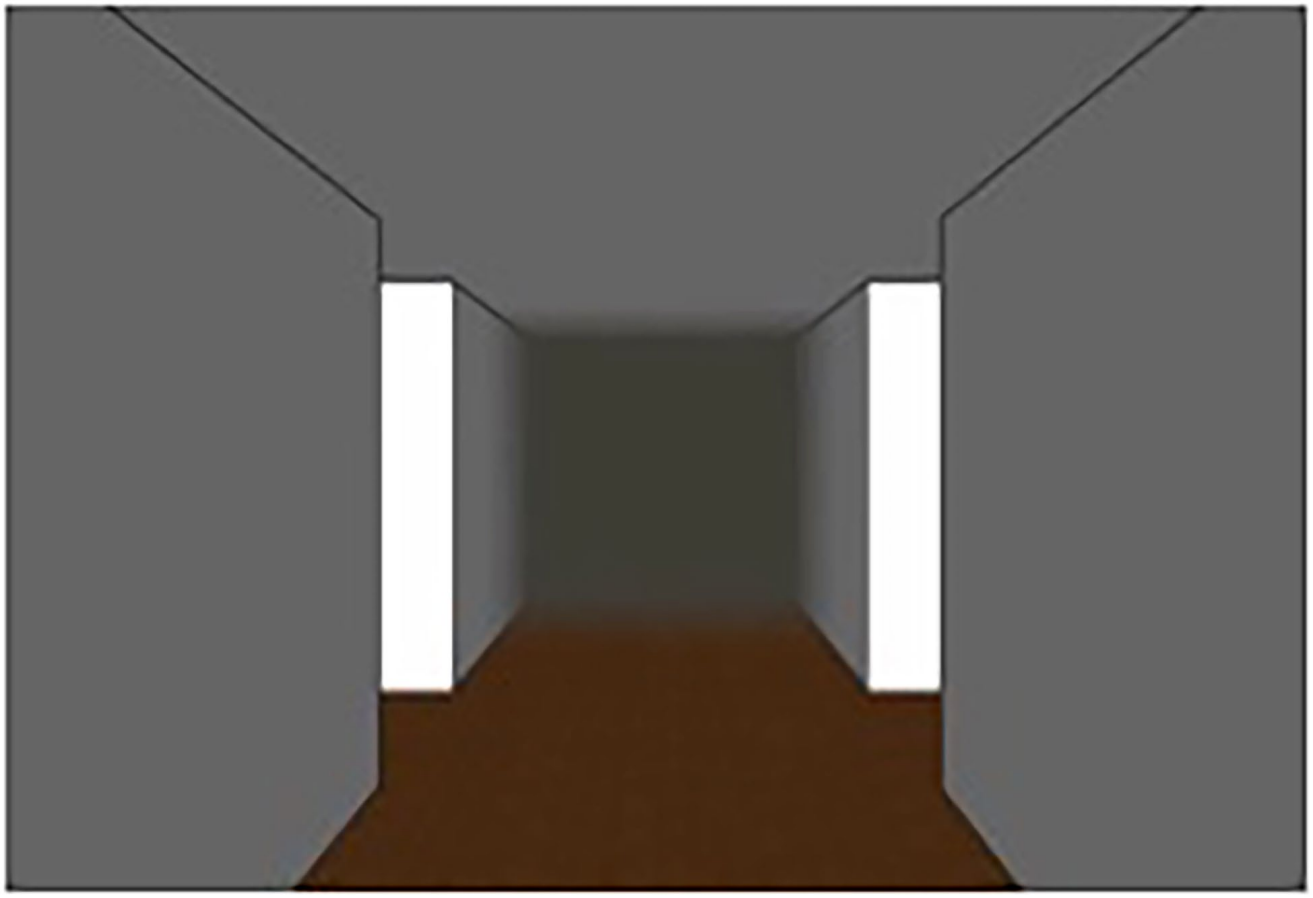
Screenshot of an (empty) intersection taken from Hamburger and Röser (2011, 2014).

For the creation of the routes, a screenshot of an empty intersection taken from two studies by Hamburger and Röser (2011, 2014) was used (see Figure 1). Furthermore, for the purpose of the study, additional images and the corresponding odor samples were required. The odors were taken from the study by Hamburger and Knauff (i.e., garlic, strawberry, cinnamon, aftershave, etc.; for further details, such as an evaluation of the odors, see Hamburger and Knauff, 2019). The odors used were those with the highest identification rates from a set of 44 odors. Odors were stored in amber glass vials. Since the odors and the images should match, images of objects matching the above odors were taken with a Samsung NX1000 SLR camera. Since participants were presented with either visual or olfactory landmark information, either images of objects implemented in the screenshot (visual landmark condition) or the screenshot of an empty intersection (Figure 1) only (olfactory landmark condition) were presented to the participants. The visual landmarks were placed in the center of the upper half of the virtual room to give the impression that the image was hanging on the ceiling of the intersection in front of the participant. If the participant was assigned to the olfactory condition, she was presented with an odor manually by the experimenter instead of the visual landmark while looking at the empty intersection. The sequence was randomized in advance.

In addition, a self-generated light gray arrow was inserted at each intersection (in the visual as well as the olfactory landmark condition) to indicate the direction. The arrow was also located in the center, but in the lower half of the virtual space. The direction in which the arrow pointed at each intersection was also pseudo-randomized.

The experiment was run on an Acer Aspire V17 Nitro with a 7th generation IntelCore i7 processor (16GB RAM). The laptop was connected to a Samsung 74-inch 4 K LED flat screen *via* HDMI. A large screen was deliberately chosen to make the environment more realistic and to ensure a stronger immersion effect. Participants provided their decisions using the numeric keypad of an external computer keyboard (1 = left, 2 = straight ahead, 3 = right).

### Procedure

**Figure 2:**
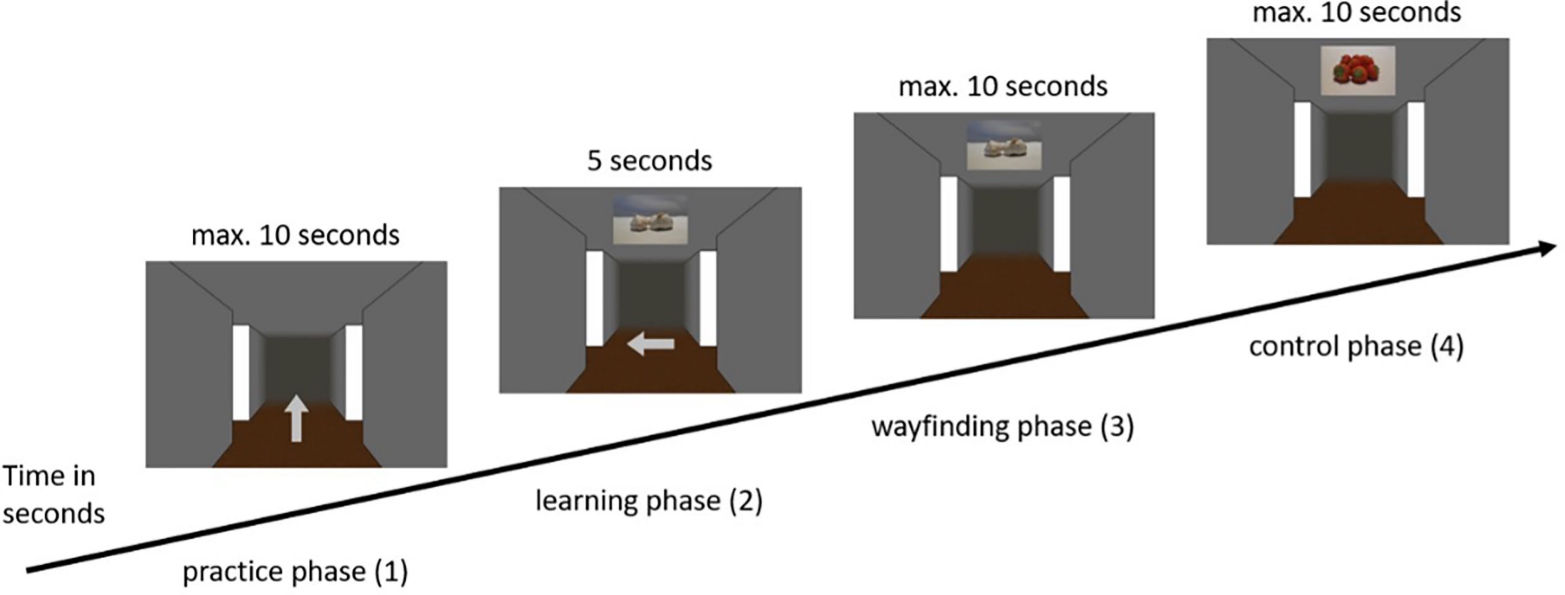
Example sequence of the main phases of the experiment in the visual condition (visual ➔ visual).

Upon arrival participants were asked to sit at a table at the end of the room, where the screen was placed. The distance between the participants and the TV was approximately 60 cm. The only thing that was varied was that the computer keyboard in front of the test person so that it was easily accessible with their hands. In addition to the informed written consent form and an instruction about the experiment, demographic data were collected. Regardless of which condition the participants were assigned to, the main experiment consisted of four phases, the practice phase (1), the learning phase (2), the wayfinding phase (3), and a randomized control phase (4). For clarification, the complete sequence of the main phases is visualized in Figure 2. Before each of these phases, the participants were presented with a detailed instruction, which they were asked to repeat orally in their own words to ensure that they understood the instruction. The instruction included an explanation of the duration of the experiment, the number as well as the sequence of the phases. In addition, each instruction included an explanation of the use of the numeric keypad and a reminder to both focus attention on the center of the screen and to make decisions as quickly and accurately as possible.

**Figure 3:**
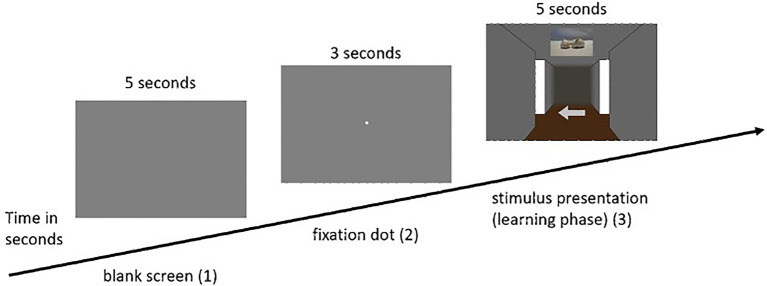
Example sequence of a single pass in the learning phase of the visual condition (visual ➔ visual).

**Figure 4:**
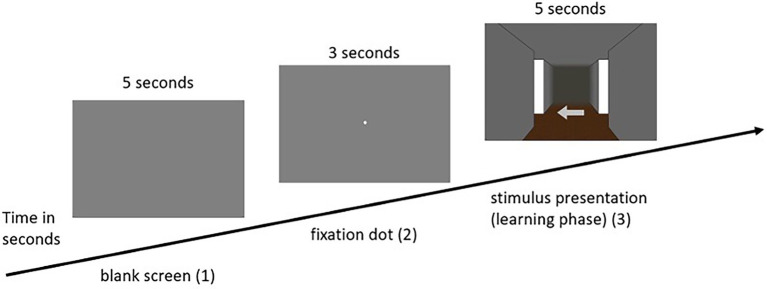
Example sequence of a single pass in the learning phase of the olfactory condition (olfactory ➔ olfactory).

(1) The first phase of the experiment was a practice phase. Each participant was led through nine trials in which she was presented only with the screenshot of the intersection with a light gray arrow in the middle. There was no presentation of visual or olfactory landmarks in the practice phase. The arrows pointed equally often either to the left, straight ahead, or to the right. Before each intersection, participants were presented with a fixation dot for 3 s to direct their attention to the following intersections. The task was to correctly respond to the presented arrow keys (1 = left, 2 = straight ahead, 3 = right) using the numeric keypad. This gave the participants the opportunity to familiarize themselves with the procedure, the virtual room and the required material, i.e., the numeric keypad of the keyboard. At the end of the practice phase, each participant was presented with feedback showing the average decision time of the trials and the average of correct route decisions in percent. The values of the feedback had no influence on the main part of the experiment that was carried out afterwards.

(2) The practice phase was followed by the learning phase, in which the participants were presented with 12 intersections. In this phase, as well as in each subsequent phase, the participants first saw a blank gray screen for 5 s, in which attention to the screen was not yet required. After that, the participants were presented with a fixation dot for another 3 s, to which the participants were asked to direct their attention. Subsequently, the respective intersection of the participant’s individually assigned route appeared. Depending on the condition assigned, participants were presented with either visual or olfactory landmark information. The task was to remember the presented landmark information with the associated direction, with each landmark (either visual or olfactory) being presented for 5 s. This procedure was based on Karimpur and Hamburger (2016), who gave the participants a maximum of 5 s to decide on directional information in a similar experiment. This was done for each of the 12 intersections. As soon as the test person had completed all 12 intersections, the learning phase ended (for an example trial of the learning phase in the visual condition see Figure 3; for the olfactory condition see Figure 4, for a schematic illustration of the wayfinding phase see also Hamburger and Knauff (2019).

(3) The next phase was the so-called wayfinding phase. Here, the participants were presented with the same route sequence as in the learning phase. The difference, however, was that in the wayfinding phase (either visual or olfactory, see Figures 3, 4) the presentation of the arrows, i.e., the directional information, was omitted. Participants in the “no switch” condition were presented with landmark information in the same modality, while participants in the “switch” condition were presented with corresponding landmark information in the other modality. Once the landmark was presented to the participant, her task was to respond with the associated direction key. In this phase, the landmark information (either visual or olfactory) was presented for a maximum of 10 s. If the participant has already made a decision before the time expired, the experiment went on without interruption and the gray screen appeared followed by the fixation dot and the next intersection. The same applied if the participant did not make the correct decision. The experiment also went on without interruption by the appearance of the gray screen followed by the fixation dot and the next intersection.

(4) The final phase of the experiment was the randomized control phase. Here, the previously learned intersections (i.e., combination of landmark and directional information) were tested again within the same modality as in the wayfinding phase, but in a randomized order. The randomization of the intersections made it possible to compare the third and fourth phase and to check whether the respondent had linked the landmarks to the directions or had learned the path sequentially. After the last phase with again 12 intersections, the main experiment was completed. The duration of the experiment was between 30 and 45 min.

## Results

### Switch vs. No Switch

For the question of whether switching costs occur when switching between visual and olfactory landmark information, the following results were obtained. Significances, as well as effect sizes, are reported. Because of the small sample size in each group the test assumption of normal distribution was not given for all groups and conditions. However, while the normal distribution assumption is theoretically important for unpaired *t*-tests, numerous studies have practically shown that *t*-tests are relatively robust to violations of normal distribution assumption (e.g., Rasch and Guiard, 2004; Wilcox, 2012). That is why, independent *t*-tests will still be reported in this study. Additionally, non-parametric Mann–Whitney-U-tests were calculated for the most important results of the study and are reported in brackets.

**Figure 5:**
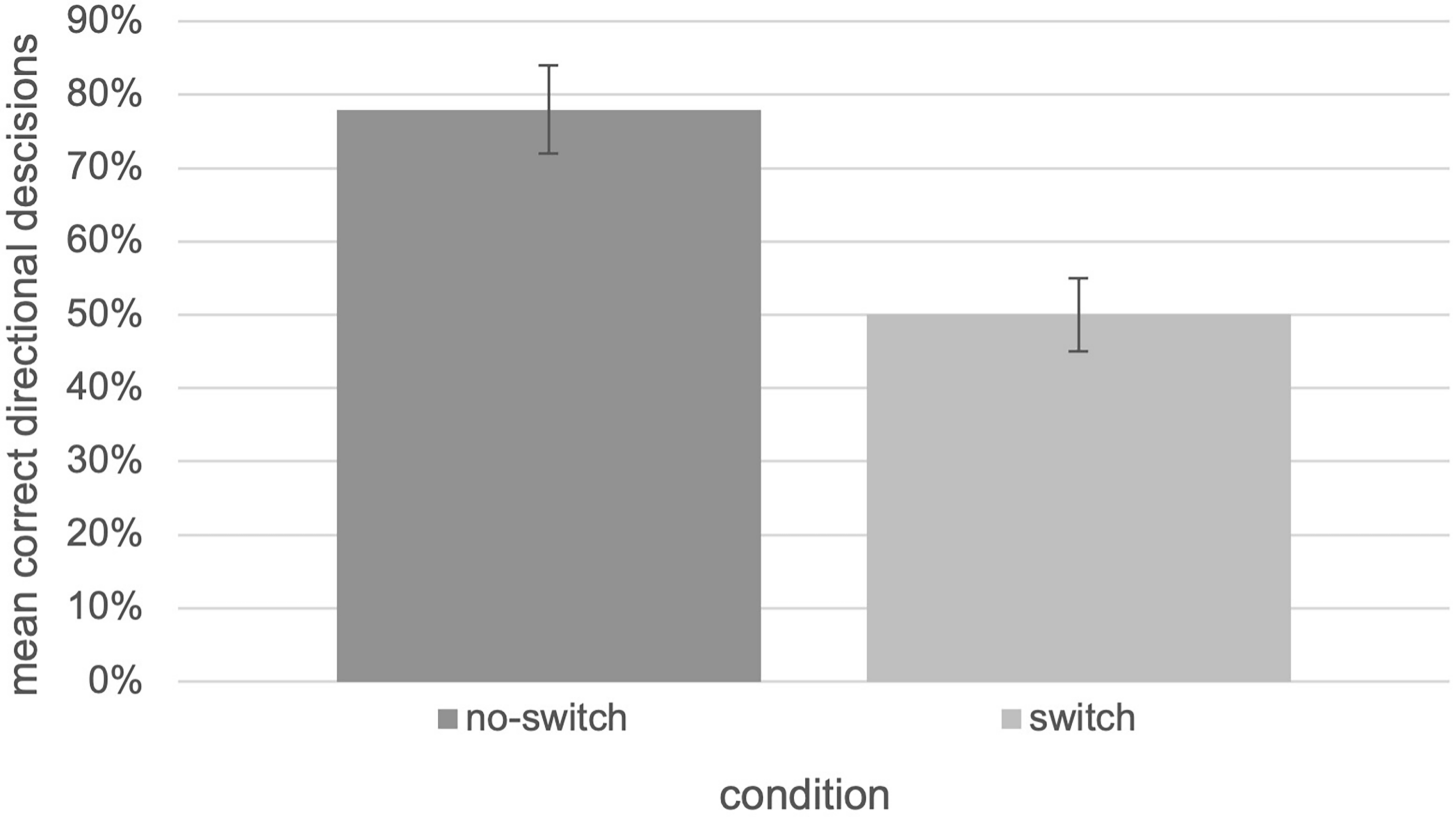
Relative number of correct decisions with respect to the “switch” and “no switch” condition of the tested experiment (*N* = 30, error bars = SEM).

Wayfinding performance, in terms of the relative number of correct decisions, for the “no-switch” condition (*M* = 0.78, SEM = 0.06) was higher than for the “switch” condition (*M* = 0.50, SEM = 0.05). These findings are visualized in Figure 5. The collected data were analyzed using an independent two-tailed t-test which revealed significant differences between the “no-switch” and “switch” condition, *t*(27.35) = 3.38, *p* = 0.002, *d* = 0.931 [*U* = 46.00, *Z* = −2.78=, *p* = 0.005; according to Cohen, 1988 effect sizes are interpreted as follows: small effect size *d* = 0.2, medium effect size *d* = 0.5, large effect size *d* = 0.8].

**Figure 6:**
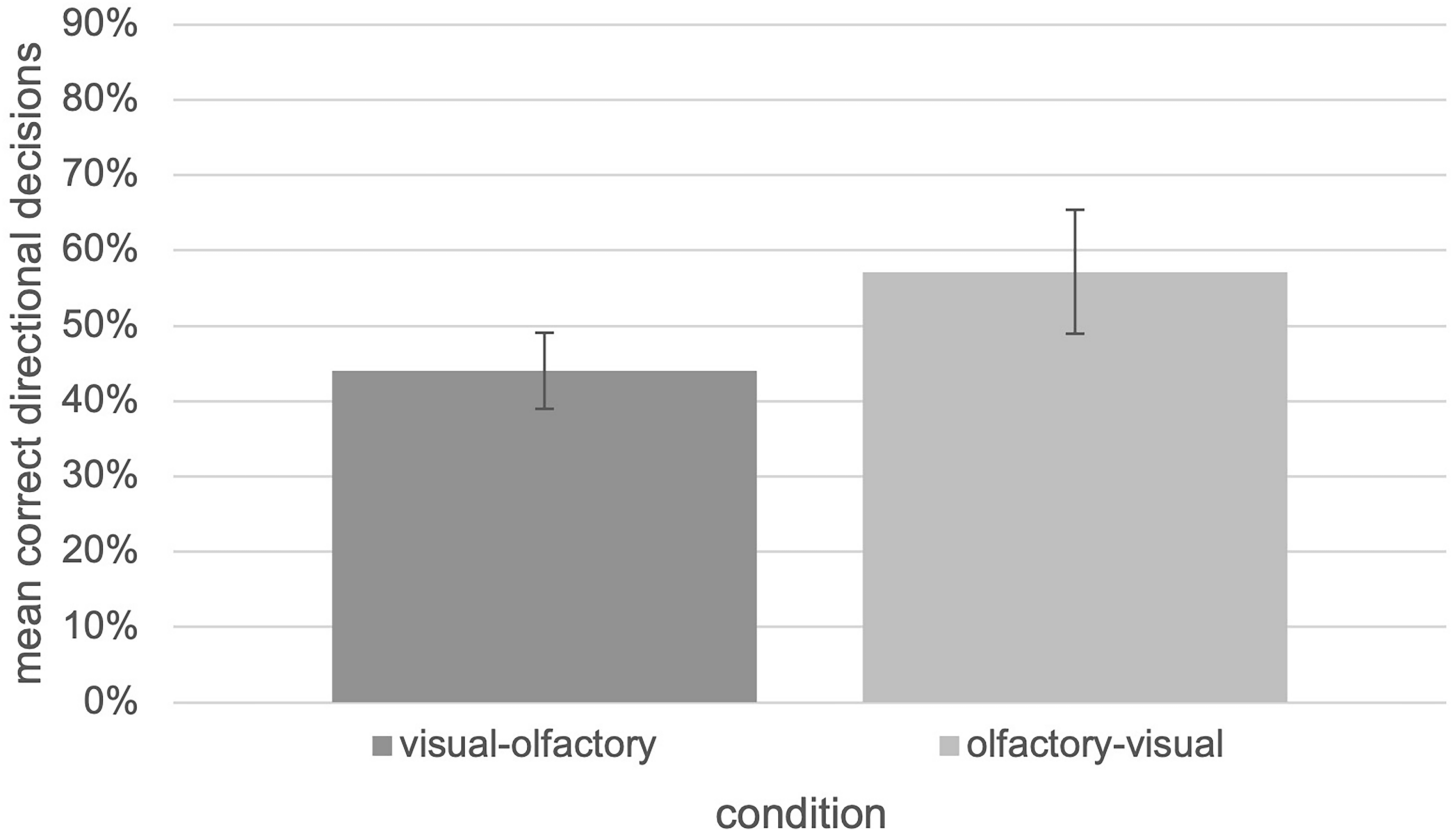
Relative number of correct decisions with respect to the “visual-olfactory” and “olfactory-visual” switch condition of the tested experiment (*N* = 30, error bars = SEM).

In general, it turns out that a modality switch between visual and olfactory landmark information is possible since performance is significantly above chance level as shown by an one-sample *t*-test, *t*(13) = 3.535, *p* = 0.004, *d* = 0.186. This result is independent of the switch-direction as an independent two-tailed *t*-test which revealed no significant differences between the “visual ➔ olfactory” and “olfactory ➔ visual” “switch” condition, *t*(12) = −1,357, *p* = 0.20, *d* = 0.180 (*U* = 15.00, *Z* = −1.236, *p* = 0.217). However, the “switch” condition seems to be associated with further cognitive costs in terms of a lower number of correct decisions (Figure 6).

**Figure 7:**
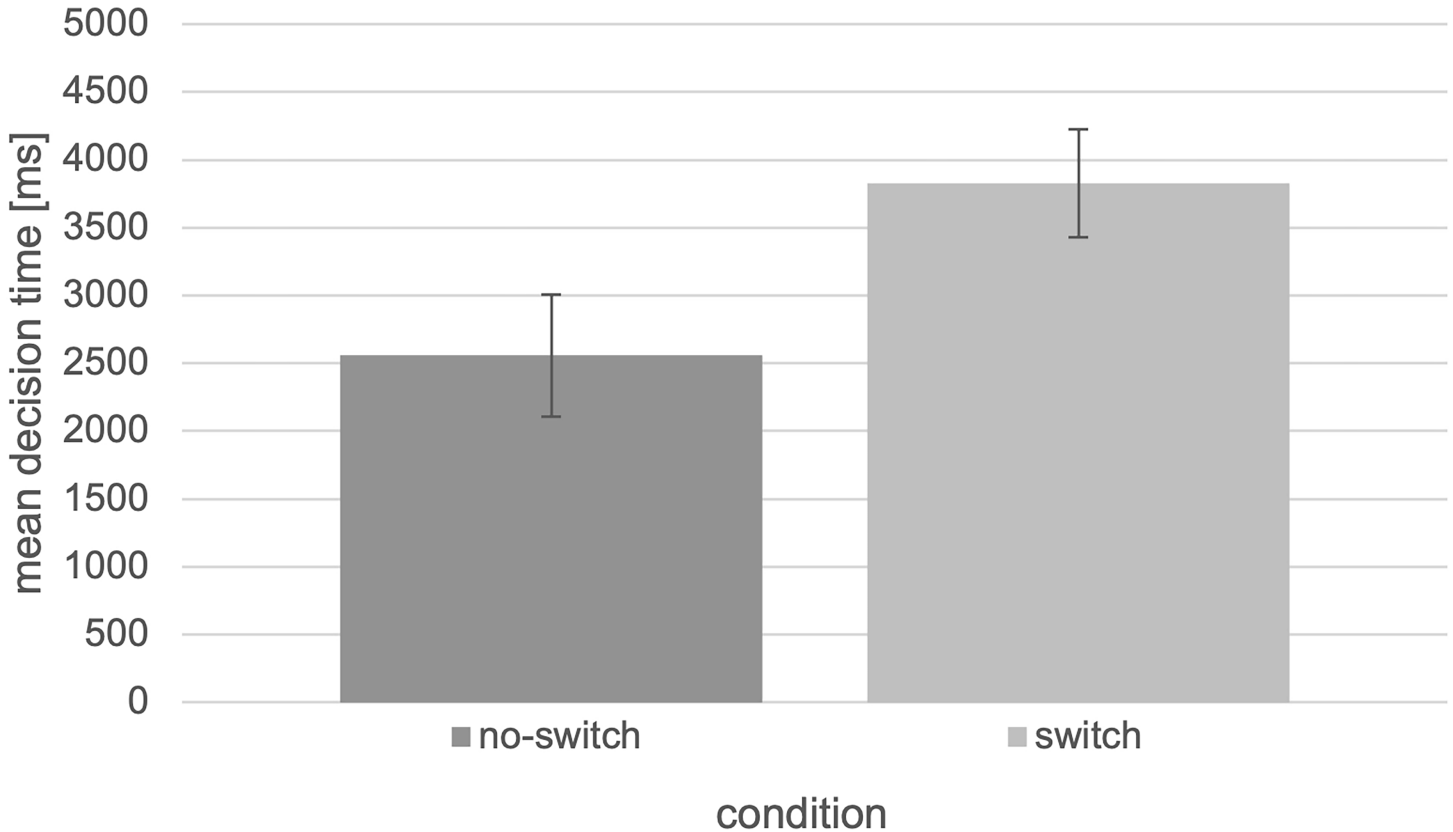
Mean decision time in ms with respect to the “switch” and “no switch” condition of the tested experiment (*N* = 30, error bars = SEM in ms).

Besides the higher performance in terms of correct decisions, there are also shorter decision times for the “no-switch” condition (*M* = 2557.70, SEM = 446.98) compared to the “switch” condition (*M* = 3827.84, SEM = 395.21; Figure 7). The collected data were also analyzed in terms of mean decision times using an independent two-tailed t-test and revealed significant differences between the “no-switch” and “switch” condition, *t*(28) = −2.10, *p* = 0.045, *d* = −0.769 (*U* = 60.00, *Z* = −2.162, *p* = 0.031).

**Figure 8:**
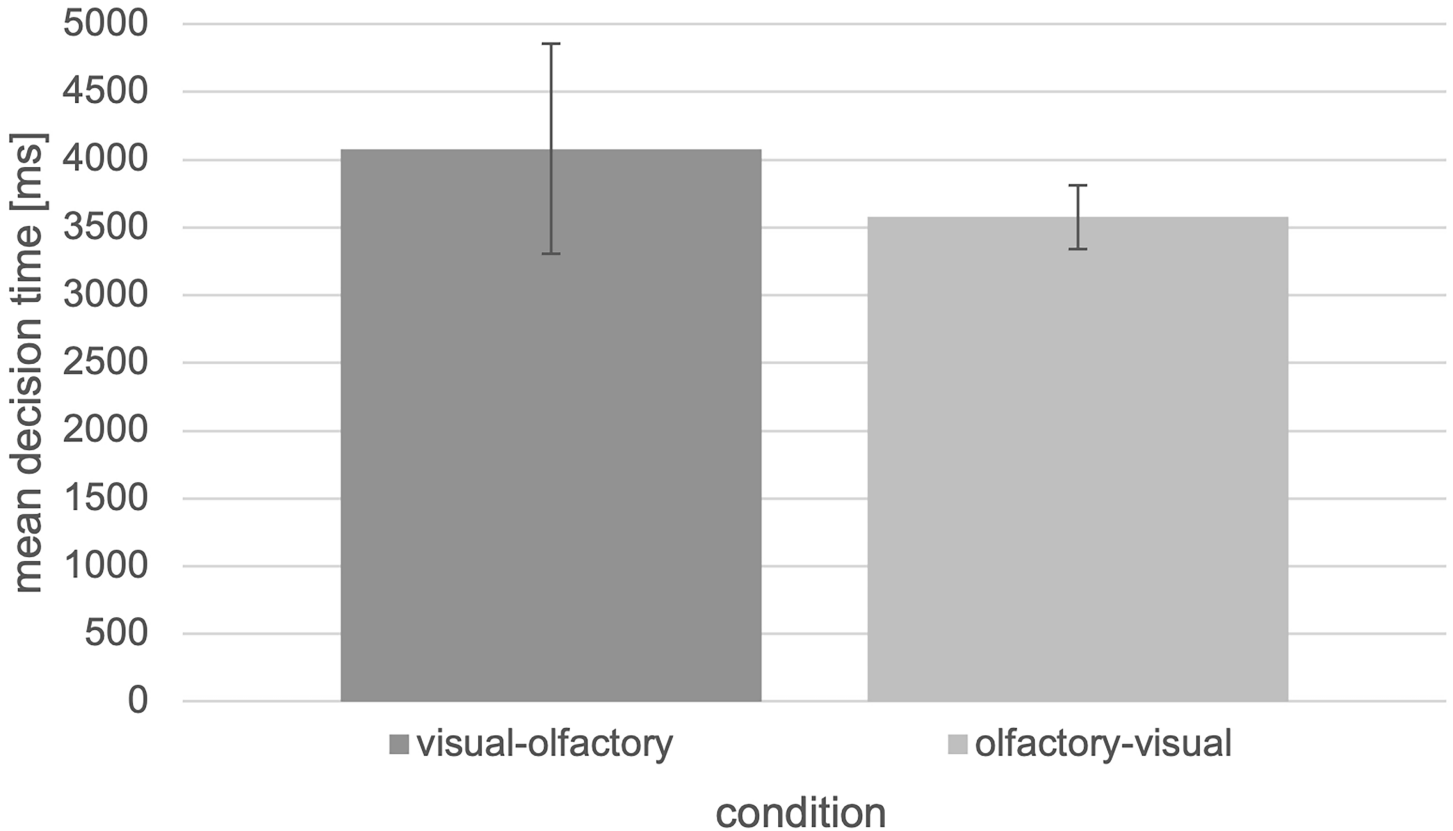
Mean decision time in ms with respect to the “visual-olfactory” and “olfactory-visual” condition of the tested experiment (*N* = 30, error bars = SEM in ms).

In this case, it is also possible to switch between olfactory and visual landmark information, but this is associated with longer decision times (Figure 8).

To test the extent to which participants oriented themselves using the landmark information and did not learn the route sequentially, a paired-samples *t*-test between wayfinding performance in terms of the relative number of correct decisions in the wayfinding phase and the control phase was conducted and showed a non-significant result, *t*(29) = 0.872, *p* = 0.391. This implies that the performance between the wayfinding phase and the subsequent randomized control phase is comparable and thus sequential learning of the route on the part of the participants can be ruled out. In the case of sequential learning, participants would have learned only the directional information without a connection to the presented landmark information, and in the case of randomized presentation of the landmark information, the control phase performance would have had to be at chance level.

### Comparison of All Levels

In addition to comparing the no-switch and switch conditions, comparisons were also made between all levels present, both for the relative number of correct decisions with *F*(3,26) = 22.425, *p* < 0.001, *f* = 1.608 [according to Cohen, 1988 effect sizes are interpreted as follows: small effect size *f* = 0.1, medium effect size *f* = 0.25, large effect size *f* = 0.4], and the mean decision times with *F*(3,26) = 10.362, *p* < 0.001, *f* = 1.094.

The comparison of the relative number of correct decisions between the visual (*M* = 0.972, SEM = 0.048) and olfactory groups (*M* = 0.524, SEM = 0.054) in the no-switch condition, showed a significant difference, *t*(26) = −6.176, *p* < 0.001, *r* = 0.771 (*U* = 0.000, *Z* = −3.440, *p* < 0.001) [according to Cohen, 1988 effect sizes are interpreted as follows: small effect size *r* = 0.1, medium effect size *r* = 0.3, large effect size *r* = 0.5]. Based on the higher relative number of correct decisions, it can be concluded that visual landmarks are better suited for wayfinding than olfactory landmark information.

The participants of the visual “no-switch” condition also seems to have a better performance (i.e., a higher number of correct decisions) in comparison with the participants of the visual-olfactory (“switch”) condition *t*(26) = −7.324, *p* < 0.001, *r* = 0.821 (*U* = 0.000, *Z* = −3.440, *p* < 0.001) and the participants of the olfactory-visual (“switch”) condition, *t*(26) = −5.520, *p* < 0.001, *r* = 0.735 (*U* = 6.00, *Z* = −2.829, *p* = 0.005).

The mean decision times of the two “no-switch” conditions visual (*M* = 1249.19, SEM = 414.90) and olfactory (*M* = 4240.07, SEM = 470.40) also differed significantly from each other, *t*(26) = 4.769, *p* < 0.001, *r* = 0.683 (*U* = 0.000, *Z* = −3.334, *p* < 0.001).

## Discussion

In general, it turns out that a modality switch between visual and olfactory landmark information is possible since performance is significantly above chance level. In contrast to the switching costs between modalities other than olfaction, a modality switch between visual landmarks and olfactory landmark information seems to be associated with further cognitive costs in terms of a lower number of correct decisions.

First, it can be said that it was possible for participants to switch between visual and olfactory landmark information in a wayfinding task. Our results imply that humans may very well use their sense of smell to orientate and navigate. According to Cunningham et al. (2021), the ability to track olfactory plumes may have been an important skill in foraging. However, this incurred additional cognitive costs, which manifested themselves in the form of a lower relative number of correct decisions and higher mean decision times. This is surprising given the empirical data for switching costs in other modalities. Since, for instance, Hamburger and Röser (2011) showed no switching costs in the performance when switching modality from auditory to visual and vice versa.

This could be explained by the fact that auditory information engages both the phonological loop and the visuospatial sketchpad of working memory (Baddeley and Hitch, 1974; Tranel et al., 2003). This would mean that sounds are also initially processed in a different modality as well, namely as images. Thus, there would be an advantage for switching between both modalities within a wayfinding task, as no additional cognitive resources would be required to transfer the learned information into the other modality. In this case it would create a facilitation effect (Hamburger and Röser, 2014), which does not seem to be the case for olfactory information. Consistent with the assumption, Hamburger and Röser (2011) found no switching costs between auditory and visual landmark information. It is possible that neither odors nor images are initially processed in the other modality, which could explain the poorer performance of the participants in the “switch” condition, as it would require additional cognitive effort to transfer the information to the other modality. On the other hand, our findings illustrated in Figure 6 also show that it was easier for the participants to switch from olfactory to visual stimuli than vice versa. This means an advantage for switching from olfactory to visual stimuli since fewer cognitive effort is required to transfer the olfactory information into the visual modality. In addition to the above explanation, based on these results, it is also possible that odors are initially processed in the visual modality as well (i.e., mental images), but images do not mentally occur in the olfactory modality, which would explain the participants’ poorer performance in the “visual to olfactory” switching condition. If this were the case, it would mean an initial double-coding for the first case but a single-coding for the second. Thus, additional cognitive resources would only be required when the visual needs to be transferred to the olfactory modality during information retrieval.

Overall, it can be concluded that humans are able to orient themselves even when switching between visual and olfactory landmark information, but their performance decreases compared to a switch between visual and auditory information.

In addition to a lower relative number of correct decisions, decision times were higher in the “switch” condition than in the “no-switch” condition, which is consistent with the hypothesis about switching costs, i.e., decision times. Although, the decision times of olfactory stimuli should not be overestimated due to ambiguous findings. Since literature shows that response times for the olfactory system are significantly longer than for visual stimuli (Cain, 1976; Spence et al., 2000), this could also explain the differences in response time that we report. On the one hand, according to Radil and Wysocki (1998), the sense of smell is a diffuse sense, which is why an exact localization of olfactory stimuli proves to be difficult. On the other hand, Porter et al. (2007) also investigated the sense of smell in humans and the ability to scent-track based on odors. According to them, humans are able to follow olfactory traces and even improve with practice.

### Limitations

It is unclear whether the presentation of olfactory stimuli also triggers increased activation in visual cortex as described above, as is the case with auditory stimuli. To investigate this further, imaging techniques would have to be utilized after the application of odors. It could then be clarified whether olfactory information is initially also processed in the visual or another modality. This could provide further insight into landmark-based wayfinding as well.

Another explanation could also be of a methodological nature. The odors presented to the participants as landmark information were presented by hand, which is the reason why no standardized presentation of the stimuli was possible. Since the focus was on the investigation of switching costs, this did not pose a problem in answering the question to be examined. However, Jacobs (2012) showed that an unequal distance of the odors to the nose results in a different intensity, which may affect the performance and decision times of the experimental participants. Therefore, for future research and especially for a time-accurate interpretation of odors, it would be useful to utilize devices that allow a standardized presentation of odors. This could be circumvented by using an olfactometer, which is capable of rapidly delivering discrete odor stimuli without tactile, thermal, or auditory variations (Gottfried et al., 2002), and which would allow a more valid interpretation of the decision times. Moreover, the presentation of odors by hand while seeing an empty intersection on screen limits the ecological validity. Since this study serves primarily as fundamental research, the focus here was on whether navigation with a modality switch is possible. Future studies, i.e., application studies, should use a more realistic implementation of the odor cues, for example by doing an open-field study where the olfactory landmark cues would be located along a real-world route.

In general, participants in all stages in which odors were included showed poorer performance compared to the participants in the condition in which only visual stimuli were tested (“no-switch”). However, it must be emphasized that this is only due to the increased difficulty of using olfactory cues in visual environments compared to using visual cues and not due to a general inability of humans to orient and navigate using olfactory landmark information, as evidenced by several studies mentioned above (e.g., Jacobs et al., 2015; Hamburger and Knauff, 2019).

In addition to the already mentioned higher initial processing times of olfactory than for visual inputs (Cain, 1976; Spence et al., 2000), the emotionality of the participants could possibly provide an explanation. Emotions generally have an influence in wayfinding as well, as demonstrated by Palmiero and Piccardi (2017). In this study participants who saw visual emotional landmark information showed better orientation performance than participants who saw neutral emotional landmarks, which is in line with the results of Balaban et al. (2017). Accordingly, an emotional association appears to have an impact on wayfinding performance when visual landmark information is presented, but whether this is also the case for olfactory landmark information is unclear. Moreover, Bestgen et al. (2015) showed that the emotional quality of odors predicts odor identification. However, it is yet unclear, whether odor quality might have an impact on (spatial) memory performance and therefore human wayfinding as well.

It is equally possible that the *Proust effect* (e.g., Van Campen, 2014) applies to olfactory landmark information. This effect occurs when odors induce episodic memories. Here, odors evoke different memories and could thus lead not only to a higher load on the cognitive system (i.e., working memory) but also to a distraction from the actual wayfinding task. Accordingly, this could likely cause longer decision times. This would mean that if a certain odor were to induce a specific memory from the past, the working memory would be under more load and an additional cognitive effort would be the result. On the one hand, the load on working memory could lead to a greater depth of processing, but on the other hand, triggered memories could also provide distraction and thus poorer performance (e.g., attention), which the results tend to suggest. Thus, the research interest extends to landmark-based wayfinding of olfactory cues with an emotional component. Specifically, we could investigate whether a specific emotional meaning of the stimuli, i.e., positive, negative, or neutral, leads to differences in orientation performance.

Closely related to this is also the salience of odors. Caduff and Timpf (2008) focused on the concept of saliency, which refers to relatively distinct, salient, or obvious features compared to other features. Visual salience dominates visual attention during indoor wayfinding (Dong et al., 2020). It is questionable whether the salience of olfactory information also influences participants’ wayfinding.

### Conclusion

With this study, we demonstrated that a modality switch between visual and olfactory landmark information has a significant impact on wayfinding. For this reason, we again underline the necessity to consider different approaches to study the role of the different modalities in landmark-based wayfinding, in order to achieve a more comprehensive understanding of the underlying cognitive processes in human spatial orientation.

## Data Availability Statement

The raw data supporting the conclusions of this article will be made available by the authors, without undue reservation.

## Ethics Statement

The studies involving human participants were reviewed and approved by FB06, JLU Giessen; 2014-0017. The participants provided their written informed consent to participate in this study.

## Author Contributions

KH contributed to conception and design of the study and organized the database. KH and MS performed the statistical analysis. MS wrote all sections of the manuscript. All authors contributed to the article and approved the submitted version.

## Conflict of Interest

The authors declare that the research was conducted in the absence of any commercial or financial relationships that could be construed as a potential conflict of interest.

## Publisher’s Note

All claims expressed in this article are solely those of the authors and do not necessarily represent those of their affiliated organizations, or those of the publisher, the editors and the reviewers. Any product that may be evaluated in this article, or claim that may be made by its manufacturer, is not guaranteed or endorsed by the publisher.
